# Characterization of Novel POLG Mutations in Mitochondrial Encephalomyopathy: Pathogenic Validation and Comprehensive Genetic Profiling

**DOI:** 10.1002/brb3.71045

**Published:** 2025-11-11

**Authors:** Fanjing Zhou, Jiang Chen, Tingzheng Zhang, Fengnan Niu, Jinglong Hu, Yun Xu, Meijuan Zhang

**Affiliations:** ^1^ Department of Neurology, Nanjing Drum Tower Hospital, Affiliated Hospital of Medical School Nanjing University Nanjing China; ^2^ Department of Neurology, Nanjing Drum Tower Hospital, State Key Laboratory of Pharmaceutical Biotechnology and Institute of Translational Medicine for Brain Critical Diseases Nanjing University Nanjing China

**Keywords:** mitochondrial encephalomyopathy, POLG mutation, SANDO

## Abstract

**Introduction/Aims:**

Mitochondrial encephalomyopathies are multisystem disorders caused by defects in mitochondrial DNA (mtDNA) or nuclear DNA (nDNA). Sensory ataxic neuropathy, dysarthria, and ophthalmoparesis (SANDO) syndrome is a rare manifestation, often associated with POLG mutations. This study identifies a novel POLG mutation in a SANDO patient, validates its pathogenicity, and analyzes the molecular genetics of 61 reported POLG‐SANDO cases.

**Methods:**

After obtaining informed consent, the proband underwent neurological examination, electromyography, muscle/nerve biopsies (histochemical/ultrastructural analyses), and genetic testing (whole‐exome sequencing, mtDNA analysis). Pathogenicity of identified POLG variants was assessed in Cas9‐mediated primary neuronal models expressing mutant proteins by measuring reactive oxygen species (ROS) levels and mtDNA copy number (qRT‐PCR, ND1/APP ratio). Literature searches (PubMed, CNKI, Wanfang, and ClinVar) identified reported POLG mutations and clinical features in SANDO.

**Results:**

Clinical and biopsy findings confirmed SANDO syndrome. Genetic analysis revealed compound heterozygous POLG mutations: a novel c.3297G>C (p.W1099C) and a known c.1774C>T (p.L592F). Neurons expressing either mutant exhibited elevated ROS levels (*p* < 0.05) and reduced mtDNA copy number compared with controls. Literature synthesis identified over 30 SANDO‐associated POLG mutations, with p.A467T (31.2%) and p.W748S (22.1%) being the most frequent. The mean age of onset was 31.6 years.

**Conclusions:**

We identify a novel pathogenic POLG variant (p.W1099C) causing mitochondrial dysfunction via impaired mtDNA maintenance, expanding the SANDO genetic spectrum. Functional studies confirmed both mutations induce mitochondrial dysfunction (elevated ROS and decreased mtDNA Copy Number), validating their pathogenicity. The compiled mutation profile aids diagnosis of this phenotypically heterogeneous, frequently misdiagnosed disorder.

## Graphical abstract

We report a 32‐year‐old female patient with SANDO syndrome (a subtype of mitochondrial encephalomyopathy), who carries two heterozygous POLG mutations: a novel c.3297G>C and a known c.1774C>T. The neuronal models expressing these mutations demonstrated mitochondrial dysfunction, thereby confirming their pathogenicity. Additionally, a summary of POLG mutations related to SANDO is provided to facilitate clinical diagnosis.

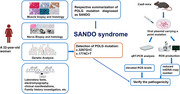

## 1 Introduction

Mitochondrial diseases are hereditary metabolic disorders caused by mutations in mitochondrial DNA (mtDNA) or nuclear DNA (nDNA), leading to dysfunction of the mitochondrial respiratory chain. These diseases present with a variety of clinical manifestations. Subtypes of mitochondrial encephalomyopathy include Kearns–Sayre syndrome (KSS), chronic progressive external ophthalmoplegia (CPEO), myoclonus epilepsy with ragged‐red fibers (MERRF), mitochondrial encephalomyopathy with lactic acidosis and stroke‐like episodes (MELAS), sensory ataxic neuropathy, dysarthria, and ophthalmoparesis (SANDO), and so on (Rahman and Copeland 2019).

The mtDNA polymerase (Pol γ) is the only replicative enzyme in mitochondria, encoded by the autosomal POLG gene. Pol γ is a nuclear‐encoded protein that has both polymerase activity and a proofreading exonuclease activity on mtDNA (Longley et al. 2001). Therefore, POLG gene mutations lead to a severe decline in the catalytic ability of DNA Pol γ and the occurrence of mitochondrial diseases, which is similar with the phenotypes as patients with inherited mtDNA mutations (Cohen et al. 1993). The phenotypic spectrum of POLG‐related disorders is heterogeneous, making clinical diagnosis challenging. Here, we describe a patient presenting with sensory ataxia, dysarthria, and ophthalmoparesis, diagnosed as SANDO syndrome associated with two heterozygous variants in POLG: c.3297G>C (p.W1099C) and the novel c.1774C>T (p.L592F). We confirmed the pathogenicity of this mutation by a combination of methods, including clinical evaluation, muscle histochemistry, nerve histochemistry, and assessment of mitochondrial ultrastructural alterations. Furthermore, we established murine neuronal models harboring these specific point mutations. By assessing mtDNA copy number and reactive oxygen species (ROS) levels in these models, we further demonstrated that the mutations resulted in mitochondrial dysfunction.

To contextualize our findings, we searched the literature on POLG‐related SANDO and reviewed previously reported cases to summarize the clinical and genetic features of this disorder.

## 2 Materials and Methods

### 2.1 Muscle Biopsy and Histology

A Biceps brachii muscle specimen was snap‐frozen in liquid nitrogen‐cooled isopentane. Transverse cryosections (8 µm thickness) were cut according to muscle fiber orientation. The sections were subjected to histological staining with Hematoxylin and Eosin (HE), Modified Gomori Trichrome (MGT), Periodic Acid‐Schiff (PAS), Succinate Dehydrogenase (SDH), Cytochrome c Oxidase (COX), and Oil Red O (ORO).

### 2.2 Sural Nerve Biopsy and Histology

Under local anesthesia, the sural nerve was exposed via a longitudinal incision, dissected at its proximal and distal ends, and injected with anesthetic near the proximal stump prior to dissection to minimize procedural pain. A segment of sural nerve tissue (grayish‐white, approximately 0.8 × 0.3 × 0.2 cm^3^) was fixed in neutral buffered formalin for light microscopic examination, including histochemical staining (HE, toluidine blue) and immunohistochemistry (myelin basic protein [MBP], neurofilament [NF], CD4, CD8, CD20).

In addition, a separate segment (cord‐like, grayish‐yellow, approximately 0.9 × 0.4 × 0.3cm^3^) was fixed in glutaraldehyde for ultrastructural examination by transmission electron microscopy.

### 2.3 Genetic Analysis

Peripheral blood samples were collected from the proband and her parents after obtaining written informed consent. Pathological dynamic mutations in seven spinocerebellar ataxia (SCA) subtypes (SCA1, SCA3, SCA6, SCA7, SCA12, and DRPLA) were screened at KingMed Diagnostics (Guangzhou, China) using fluorescent PCR‐based fragment analysis and capillary electrophoresis.

Whole‐exome sequencing (WES) was performed using the Verita Trekker variant detection system and Enliven variant annotation and interpretation system, both independently developed by Berry Genomics (Beijing, China). Suspicious WES findings indicative of dynamic mutations were further validated using PCR followed by capillary electrophoresis analysis.

mtDNA was analyzed by bidirectional Sanger sequencing, specifically targeting nucleotide positions 3243, 3252, and 3271 within the MT‐TL1 gene, and positions 8344, 8356, 8361, and 8363 within the MT‐TK gene. The mtDNA sequence NC_012920.1 from the NCBI database served as the standard reference sequence. Sequence data obtained from both peripheral blood and urine sediment cell samples were compared against this reference sequence to identify pathogenic variants associated with mitochondrial encephalomyopathy.

### 2.4 Mice and Cell Culture

Cas9 mice (*Rosa26‐CAG‐spCas9*, body weight 25–28 g) were provided by the Animal Center of Nanjing Drum Tower Hospital. The mice were bred and housed in an air‐conditioned, temperature‐controlled (20°C–26°C), and humidity‐controlled (40%–70%) room under a 12‐h light/dark cycle. All animal experiments were conducted under the guidelines of the Animal Use and Care Committee of Nanjing University. Cortical neurons were isolated from embryonic Day 13.5 (E13.5) fetuses for primary culture. Neurons were randomly divided into four groups and transfected the cells with each of the five lenti‐viruses (LVs, produced by OBio Tech, China):

All the viruses were constructed based on the empty vector: LV‐UbC‐WPRE‐bGH PolyA (UbC promoter is used to ubiquitously express the aimed molecule, WPRE is an element to enhance mRNA expression, and bGH PolyA mediates transcript termination).

**mPolg KO**: LV‐U6‐mPolg sgRNA‐UbC‐WPRE‐bGH PolyA (mPolg, mouse *Polg* gene, MGI:1196389. U6 promoter was used to express the sgRNA. sgRNA target sequence, 5’‐ GGCGGCCGCCTCGTTATTGG‐3’)

**mPolg KO + WT Rescue**: LV‐U6‐mPolg sgRNA‐UbC‐mPolg(sgRes)_3 × HA‐WPRE‐bGH PolyA (mPolg CDS, NCBI_ CCDS21382.1. c.421T>C, c.423G>A, and c.426G>A mutations were introduced into the CDS for sgRNA resistance (sgRes) without changing the amino acid sequence. 3 × HA tag was added at the C‐terminus to label the heterogeneous mPolg protein).

**mPolg KO + L572F**: LV‐U6‐mPolg sgRNA‐UbC‐mPolg_L572F(sgRes)_3 × HA‐WPRE‐bGH PolyA (c.1717C>T was introduced to obtain mPolg_L572F mutation protein, the mouse Polg mutation mPolg_L572F corresponds to the human hPOLG_L592F mutation).

**mPolg KO + W1077C**: LV‐U6‐mPolg sgRNA‐UbC‐mPolg_W1077C(sgRes)_3 × HA‐WPRE‐bGH PolyA (c.3231G>C was introduced to obtain mPolg_W1077C mutation protein, the mouse Polg mutation mPolg_W1077C corresponds to the human hPOLG_W1099C mutation.).

### 2.5 Quantitative Real‐Time PCR (qRT‐PCR) Analysis for mtDNA Copy Number

DNA was extracted using a Beyotime RNA/DNA Isolation Kit according to the manufacturer's protocol. Quantification of mtDNA copy number was performed using qRT‐ PCR. The ND1 gene fragment of the mitochondrial genome was amplified from all the individuals using the primer ND1‐F (5’‐ CCCTAAAACCCGCCACATCT‐3’) and ND1‐R (5’‐GAGCGATGGTGAGAGCTAAGGT‐3’). The APP gene fragment of the nuclear genome was amplified from all the individuals using the primer APP‐F (5’‐TGTGTGCTCTCCCAGGTCTA‐3’) and APP‐R (5’‐CAGTTCTGGTCACTGG‐3’). qRT‐PCR was performed with an initial denaturation step of 95°C for 30 s, then 95°C denaturation for 3 s, followed by primer and probe hybridization and DNA synthesis at 60°C for 30 s; the last two steps were repeated for 40 cycles. qRT‐PCR was performed using a Step One Plus PCR system (Applied Biosystems, USA).

### 2.6 Intracellular ROS Production

Cells were incubated with 10 µM 2',7'‐dichlorodihydrofluorescein diacetate (DCFH‐DA, Beyotime) diluted in serum‐free medium for 20 min at 37°C. Briefly, the DCFH‐DA stock solution (30 mM) was diluted 1:1000 in serum‐free medium to achieve the working concentration. After removing the culture medium, the diluted DCFH‐DA solution was added. Cells were then washed three times with serum‐free medium to remove excess probe. Finally, the slides were imaged using a confocal fluorescence microscope (Olympus FV3000) and analyzed using ImageJ software (NIH).

## 3 Results

### 3.1 Patient Characterization

A 32‐year‐old woman was referred to us because of exercise intolerance, weakness, and muscle wasting. She was born 3 months prematurely. Since the first year of life, she had difficulty walking fast and was prone to falling when she ran. There is no family history of neuromuscular disease. At 19 years of age, she first developed weakness of both legs, slow walking speed, and inability to walk in a straight line. By 20 years of age, she developed weakness of both arms and legs, then slurred speech, and eye fissures.

But 12 years later, when she was 32 years old, she developed severe dysarthria and a markedly abnormal gait. Examination revealed restricted adduction and abduction of both eyes. Thyroid function tests at this time were normal, indicating that the diagnosis of hyperthyroid peripheral neuropathy clearly could not account for the current clinical presentation. EMG revealed a predominant axonal polyneuropathy affecting both motor and sensory fibers of the upper and lower limbs, with chronic neurogenic changes in distal limb muscles and possible myopathic involvement in selected proximal upper‐limb segments (Table 1). Electroencephalography (EEG) demonstrated more frequent low to medium amplitude slow waves at 4–5 Hz in both hemispheres. CSF analysis revealed normal leukocyte count (3 cells/µL), chloride (128 mmol/L), glucose (3.0 mmol/L) levels, and protein (0.26 g/L), while showing negative results for pathogenic microbial infection.

**TABLE 1:** Electromyography motor nerve conduction velocity.

Motor conduction velocity(MCV)			
Motor nerves	Latency(ms)	Amplitude(mV)	Conduction(m/s)
**Ulnar (L)**			
Wrist	1.80	5.0↓	
Above elbow—wrist	7.02	3.6↓	53.6
**Ulnar (R)**			
Wrist	1.80	5.3↓	
Above elbow—wrist	7.31	4.1↓	52.6
**Median (L)**			
Wrist	2.98	4.4↓	
Elbow—wrist	7.45	4.3↓	51.5
**Median (R)**			
Wrist	3.00	6.3↓	
Elbow—wrist	7.47	5.8↓	52.6
**Tibial (L)**			
Ankle	Absent	Absent	
Popliteal fossa—ankle	Absent	Absent	Absent
**Tibial (R)**			
Ankle	6.72	1.03↓	
Popliteal fossa—ankle	19.9	0.21↓	30.3↓
**Peroneal (L)**			
Ankle	Absent	Absent	
Fibular head—ankle	Absent	Absent	
Tibialis anterior muscle	3.38	2.3↓	Absent

Sensory conduction velocity(SCV)			
Sensory nerves	Peak latency	Amplitude(mV)	Conduction(m/s)
**Ulnar (L)**			
Digit V—wrist	Absent	Absent	
**Ulnar (R)**			
Digit V—wrist	Absent	Absent	
**Median (L)**			
Digit II—wrist	Absent	Absent	
**Median (R)**			
Digit II—wrist	Absent	Absent	
**Superficial peroneal (L)**			
Leg lateral—ankle	Absent	Absent	
**Superficial peroneal (R)**			
Leg lateral—ankle	Absent	Absent	

F‐waves studies		
Nerve	F‐latency (ms)	F(%)
**Ulnar (L)**	30.6↑	92.9
**Ulnar (R)**	31.9↑	95.0
**Median (L)**	Absent	Absent
**Median (R)**	Absent	Absent
**Tibial (L)**	Absent	Absent
**Tibial (R)**	Absent	Absent

Lactate loading test results were as follows: resting state lactate: 4.2 mmol/L; immediately after exercise: 7.1 mmol/L; and 10 min after exercise: 5.2 mmol/L. In our hospital lab, the normal range of lactate is 0.7–2.1 mmol/L.

**FIGURE 1:**
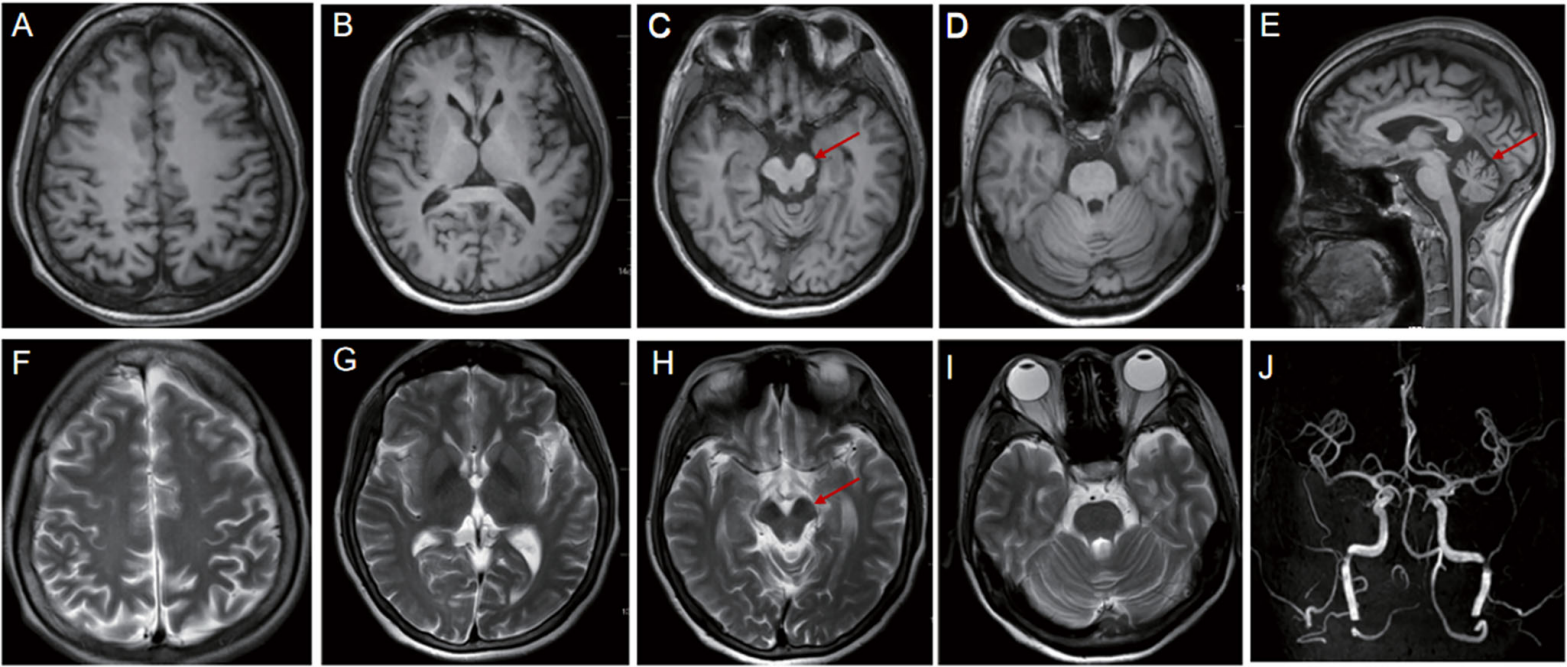
The brain MRI findings of the proband. (A) Transverse plane T1‐weighted (T1W) image at the level of the centrum semiovale. (B) Transverse plane T1W image at the level of the basal ganglia. (C) Transverse plane T1W image at the level of the midbrain. The arrow indicates midbrain atrophy. (D) Transverse plane T1W image at the level of the pons. (E) Sagittal plane T1W image. Arrow indicates cerebellar atrophy. (F) Transverse plane T2‐weighted (T2W) image at the level of the centrum semiovale. (G) Transverse plane T2W image at the level of the basal ganglia. (H) Transverse plane T2W image at the level of the midbrain. Arrow indicates midbrain atrophy. (I) Transverse plane T2W image at the level of the pons. (J) Magnetic resonance angiography (MRA) showing no significant abnormalities.

Cranial magnetic resonance imaging (MRI) showed atrophy of the midbrain and cerebellum (Figure 1). Screening for common mtDNA mutation sites (covering 80%–90% of frequent mutations associated with MELAS and MERRF syndromes) revealed no abnormalities. To further rule out the possibility of hereditary SCA, we conducted genetic testing for (SCA1, SCA2, SCA3, SCA6, SCA7, SCA12, and DRPLA), and the result was also negative. A timeline detailing the changes in clinical symptoms and corresponding examinations/treatments is provided in Table 2.

**TABLE 2:** The timeline of the patient's diagnosis and treatment.

Age (years)	Key symptoms and signs	Investigations	Treatment and outcome
**Since childhood**	Trouble running, falls often. Daily life is not affected.	Did not seek medical attention.	——
**19**	Leg weakness, unsteady walk; Slurred speech; Droopy eyelids; Normal eye movement; Decreased superficial and deep sensation; Absent reflexes; Romberg(+); Enlarged thyroid.	**Labs**: High FT3/FT4; Low TSH; Positive TRAb; Normal CK, high CKMB:31.2 U/L **Thyroid ultrasound**: diffusely enlarged; **EMG**: Suggested widespread nerve damage; **CSF: **Normal; **ECG/Chest CT**: Normal.	**Dx**: Hyperthyroidism with hyperthyroid‐associated peripheral neuropathy. **Rx**: Methimazole, Mecobalamin, Vitamin B1, B6 for nerve support. **Outcome**: Mild improvement in strength, but with persistent leg weakness, unsteady gait, and slurred speech.
**32**	Weakness of limbs, unsteady walk, inability to hold objects; Abnormal gait; Slurred speech; Droopy eyelids; Limited eye movement; Decreased superficial and deep sensation; Absent reflexes; Romberg(+); Enlarged thyroid;	**Labs: **Normal thyroid function; High CK (346 U/L), CK‐MB: 24.7 U/L; **Thyroid ultrasound**: diffusely enlarged; **EMG**: Neurogenic damage in distal muscles; possible myogenic damage in some proximal muscles; **CSF: **Normal; **ECG/Chest CT**: Normal; **MRA**: Normal brain MRA; **Neostigmine Test**: Negative; **EEG**: Mild generalized abnormality (moderate 4–5 Hz θ waves); **NCS**: Peripheral neuropathy (axonal damage, motor/sensory fibers affected); **Muscle Biopsy**: Suggested mitochondrial myopathy; **Nerve Biopsy**: Severe peripheral neuropathy; decreased myelinated fibers; **Genetics**: No common mtDNA mutations; SCA subtypes normal; **WES** revealed a point mutation in the nuclear‐encoded POLG gene.	**Dx**: SANDO syndrome. **Rx: **Mitochondrial cocktail (Idebenone, CoQ10, Vitamins B/E); Exercise therapy. **Outcome**: The patient showed slight improvement at discharge but was unable to work. Weakness continued to progress, and swallowing difficulty developed after 6 months. No effective treatment was available.

Abbreviations: CSF, cerebrospinal fluid; Dx, diagnosis; EEG, electroencephalography; EMG, electromyography; FT3, free triiodothyronine; FT4, free thyroxine; Labs, laboratory tests; NCS, nerve conduction study; Rx, treatment / prescription; TSH, thyroid‐stimulating hormone.

### 3.2 Neural and Muscle Biopsy Results

**FIGURE 2:**
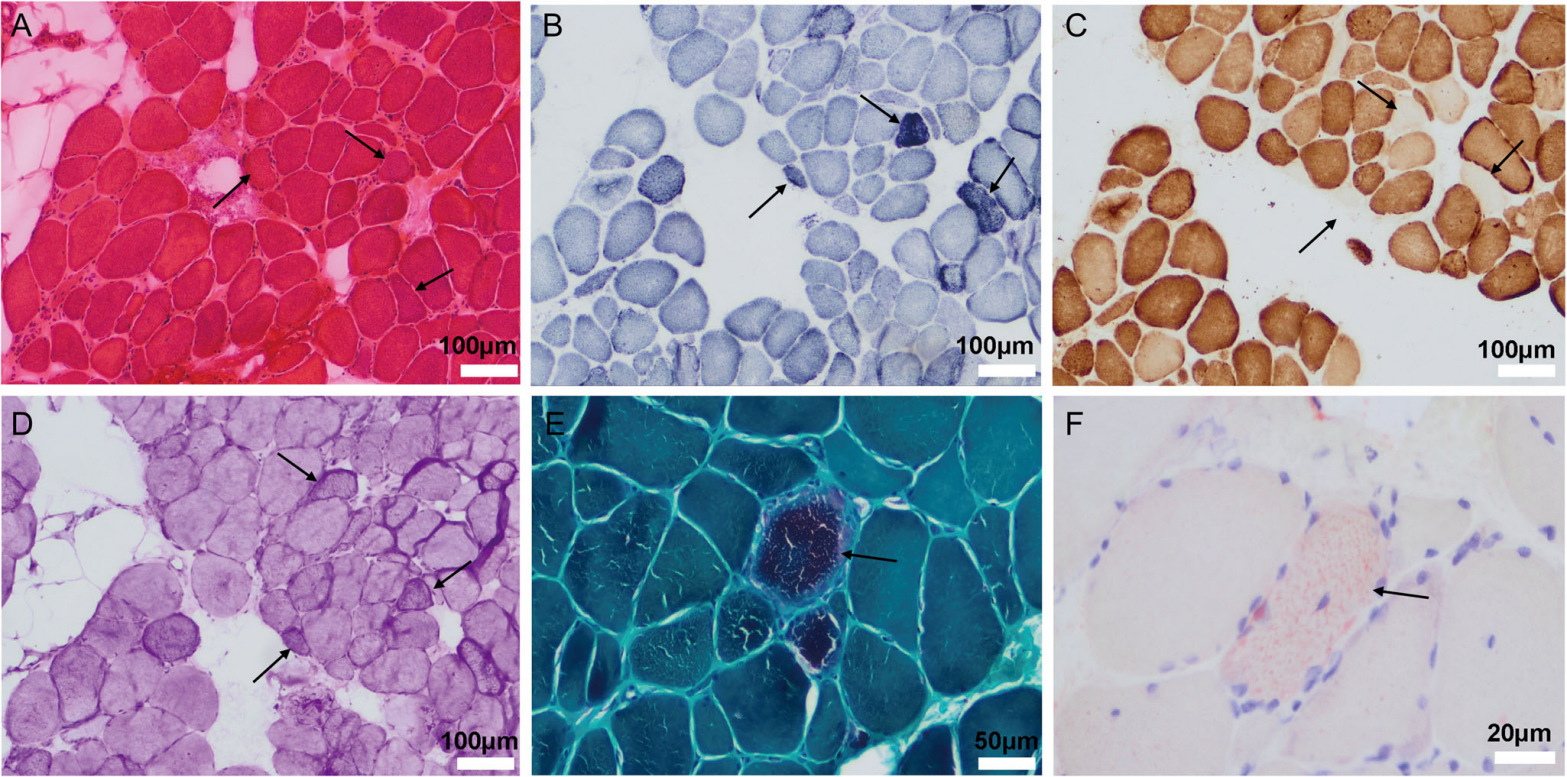
Histochemical staining of skeletal muscle sections from the patient. (A) Hematoxylin and eosin (HE) staining. Arrows indicate Type 2 muscle fiber atrophy. (B) Succinate dehydrogenase (SDH) staining. Arrows indicate ragged blue fibers (RBF). (C) Cytochrome c oxidase (COX) staining. Arrows indicate COX‐negative fibers. (D) Periodic acid‐Schiff (PAS) staining. Arrows indicate glycogen deposition within myofibers. Scale bar, as shown in the figure. (E) Modified Gomori trichrome (MGT) staining. Arrows indicate ragged red fibers (RRF). (F) Oil red O staining. Arrows indicate lipid droplet accumulation within myofibers.

**FIGURE 3:**
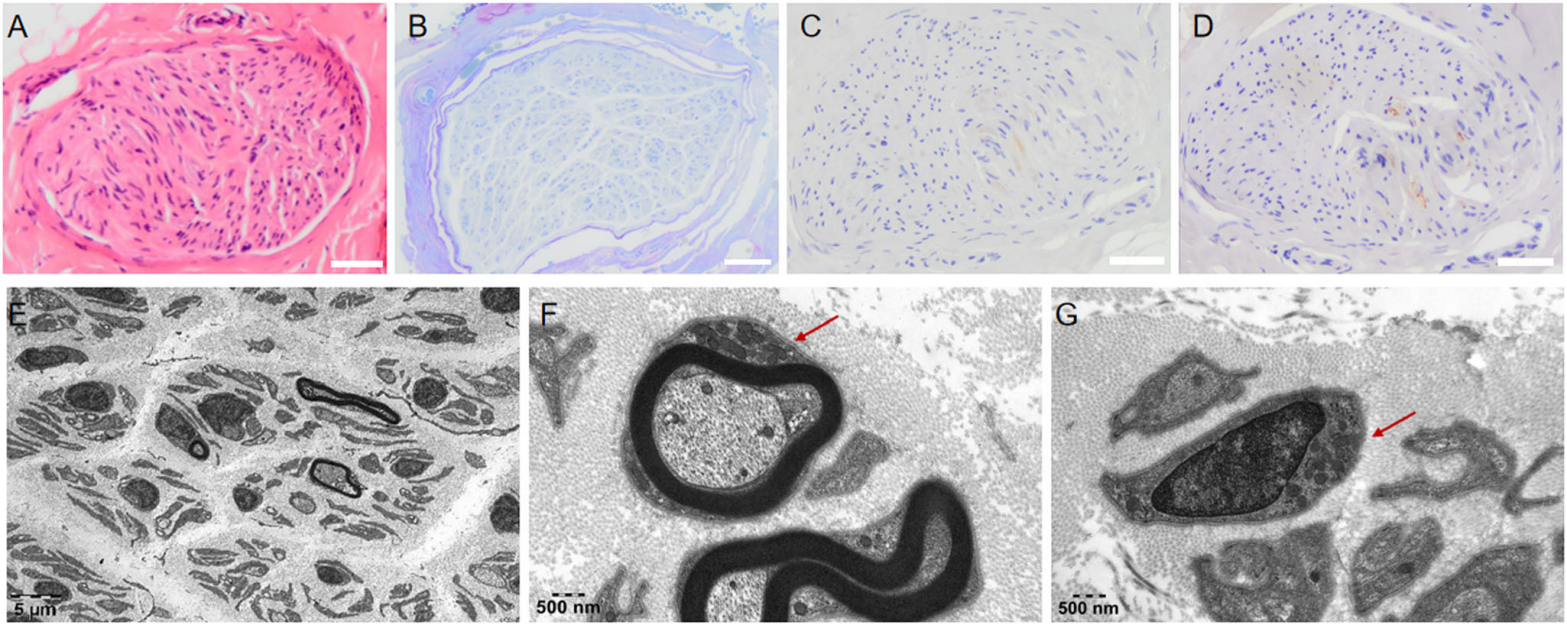
Immunohistochemical staining and electron microscopy of the patient's sural nerve. (A) Hematoxylin and eosin (HE) staining. (B) Toluidine blue staining. (C) Myelin basic protein (MBP) immunostaining. (D) Neurofilament (NF) immunostaining. (A–D) Scale bar = 50 µm. (E–G) Electron micrographs of nerve fibers. Arrows indicate mitochondrial aggregation with swollen and rounded morphology. Scale bars, as shown in the figure.

After obtaining the patient's written consent, biopsies of the left biceps brachii and sural nerve were performed under local anesthesia (Figure 2). In muscle biopsy, HE staining demonstrated Type 2 fiber atrophy. SDH staining identified ragged blue fibers, which were lightly dyed or negative by COX staining. PAS staining showed increased glycogen granules within individual muscle fibers. MGT staining revealed ragged‐red fibers, which may result from an increased mitochondrial count and morphological changes associated with abnormal mitochondrial proliferation. ORO staining indicated a mild increase in lipid droplets within scattered fibers. Immunohistochemical staining for CD4, CD8, and CD20 showed no evidence of inflammatory cell infiltration (Figure S1). In sural nerve, HE and toluidine blue staining (Figure 3A–B)showed diminished myelinated nerve fibers, reduced myelin sheaths, and sparse axons, without prominent myelin ovoids or onion‐bulb formations. MBP and NF staining (Figure 3C–D) demonstrated scanty axons within the myelinated nerve fibers in the patient's nerve tissue. Ultrastructural examination by electron microscopy (Figure 3E–G) revealed numerous swollen, rounded mitochondria accumulated within axons, and reduced nerve myelin sheath.

### 3.3 Genetic Analysis and Pathogenicity Validation

**FIGURE 4:**
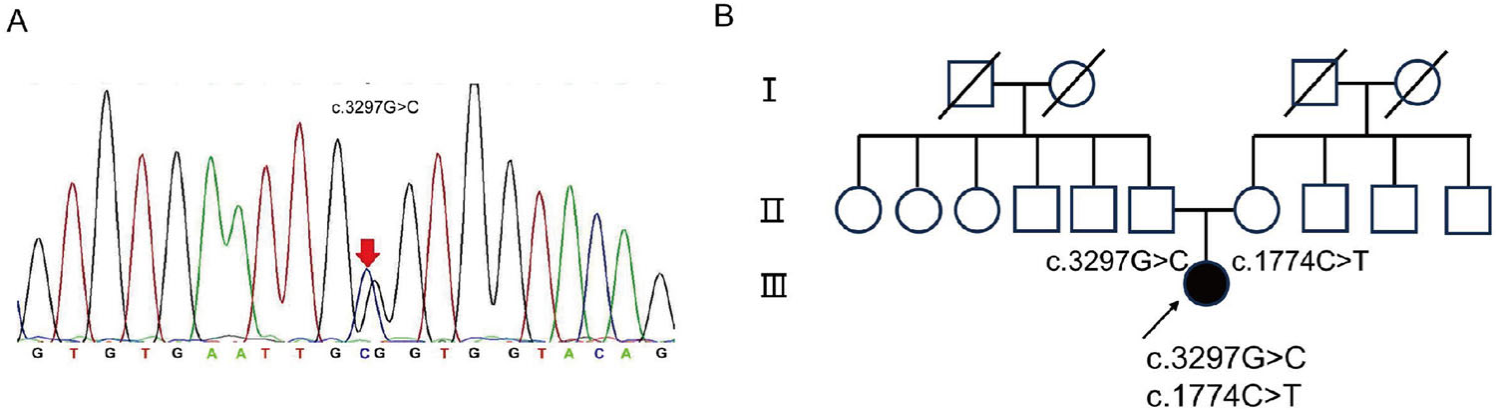
Genetic mutation identified in the proband. (A) MLPA validation results demonstrating the novel mutation site in the proband. (B) Family pedigree. Symbols marked by a slash indicate that the subject is deceased. Men are indicated by squares, women are indicated by circles. Filled circle represents the affected female proband.

Given that the patient's clinical phenotype still suggested mitochondrial myopathy, we subsequently conducted WES. WES identified three variants in the patient: a novel POLG mutation (c.3297G>C, p.W1099C) (Figure 4A), a known POLG mutation (c.1774C>T, p.L592F), and a synonymous CASQ1 variant (c.426C>T). The CASQ1 variant is a synonymous mutation inherited from the patient's mother. As synonymous mutations do not alter the amino acid sequence of the protein, they are generally considered to be benign. The mother did not exhibit any related clinical symptoms. The proband is unmarried and childless and has no siblings. We have provided the three‐generation pedigree of the proband's family, as shown in Figure 4B. No family history of similar symptoms was found.

The c.3297G>C (p.W1099C) and c.1774C>T (p.L592F) variants were absent in public population databases—including the 1000 Genomes Project, Shenzhou Genome Database, Exome Aggregation Consortium (ExAC), and Genome Aggregation Database (gnomAD)—supporting moderate pathogenicity evidence (PM2). Both variants were predicted to be deleterious by REVEL, with scores of 0.909 and 0.841, respectively (PP3_Moderate). Based on ACMG guidelines, each variant was initially classified as a variant of uncertain significance (VUS).

**FIGURE 5:**
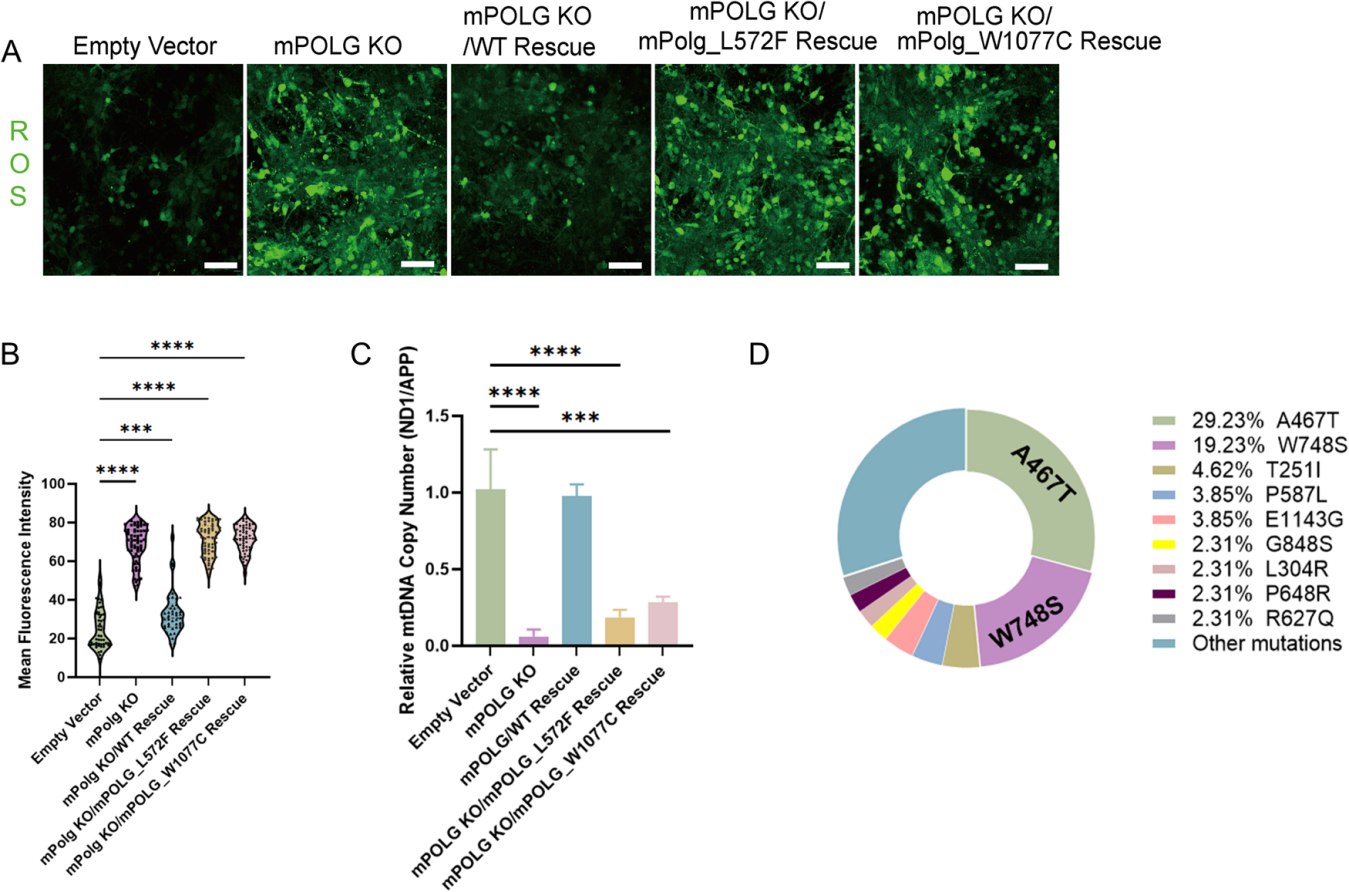
Validation of the mitochondrial dysfunction caused by the mutation site in the mutant cell model and frequency statistics of POLG mutation sites related to SANDO. (A) Representative immunofluorescence images of primary neuronal cells labeled with the ROS fluorescent probe DCFH‐DA. Scale bar = 100 µm. (B) Violin plots showing the signal intensity expression level of ROS per cell from immunofluorescence images. ***p < 0.001, ****p < 0.0001. (C) Relative mtDNA copy number was analyzed by qRT‐PCR using mitochondrial gene ND1 and nuclear gene APP and calculated by ND1/APP ratio. ***p < 0.001, ****p < 0.0001. (D) Frequency statistics of POLG mutation sites related to SANDO. The most frequently detected POLG variant associated with SANDO is the p.A467T variant (29.23% of alleles) followed by the p.W748S variant (19.23% of alleles).

To functionally validate the pathogenicity of these mutations, primary neuronal cells from the midbrain dorsal ganglia of Cas9 mice were infected with viruses harboring these mutations. Then, ROS detection and mtDNA PCR were employed to detect mitochondrial function. The efficacy of virus transfection is shown in Figure S2. Compared to empty vector and mPOLG/WT rescue groups, neurons with p.L592F mutation or p.W1077C mutation displayed profound ROS production (Figure 5A–B).

Furthermore, qRT‐PCR analysis of genomic DNA extracted from the primary neurons infected with the p.W1077C and p.W572F mutations (Figure 5C) revealed a significant reduction in mtDNA copy number (measured by the ND1/APP ratio), compared to the empty vector control group. These experimental findings provide further evidence for mitochondrial dysfunction in the mutant cell model. As a result, their classification was revised from VUS to pathogenic. These findings provide novel diagnostic insights for future patients harboring these POLG mutations, representing a key contribution of our study.

### 3.4 Respective Summarization of POLG Mutation Diagnosed as SANDO

To better understanding the mutation sites and clinical features related with SANDO, we searched previous reports from the Pubmed, CNKI, Wanfang, and ClinVar databases. There are currently over 30 reported POLG mutations that cause SANDO syndrome (Table 3). The most frequently detected POLG variant associated with SANDO is the p.A467T variant (29.23% of alleles) followed by the p.W748S variant (19.23% of alleles), with mutation frequencies detailed in Figure 5D.

**TABLE 3:** Summary of POLG mutations causing SANDO syndrome.

POLG variants	Sex and AAO (years)	Clinical features
p.F88L + p.R1096C	27F	SANDO syndrome (Li‐Xi et al. 2021).
p.L151P + p.G848A	35M	Tremor, Proximal muscle weakness, muscular atrophy, SANDO syndrome (Zhao et al. 2018).
p.N864S + p.R597Q	40M	Cataract, Preexcitation syndrome, Sensory ataxic neuropathy, ophthalmoparesis (Hanisch et al. 2015).
p.A467T + p.W748S	20F‐10‐23F‐49M‐41M‐30M‐13F‐34M‐25F	Cardiac arrest, SANDO syndrome, Deafness, Migraine, Chorea, Depression, Epilepsy, CD, Headache, Parkinsonism, Constipation, Diabetes (Kirschenbaum et al. 2018; Béreau et al. 2016; Batla et al. 2015; Bostan et al. 2012; Weiss and Saneto 2010; Richter et al. 2018).
p.T599P + p.T599P	41F	Tremor, SANDO syndrome (Gebus et al. 2018).
p.W748S + p.R627Q	48M‐38‐47	Familial amyloid polyneuropathy, CD, Depression, SANDO syndrome (Batla et al. 2015; Da Pozzo et al. 2017).
p.P648R + p.P648R	49F	Dysphagia, Cataract, Thyropathy, SANDO syndrome (Henao et al. 2016).
p.W748S + p.W748S	24F‐37‐50‐37M	Parkinsonism, Dysphagia, Migraine, CD, SANDO syndrome (Batla et al. 2015; Rouzier et al. 2014; Lovan et al. 2013).
p.A467T + p.R597W	20	SANDO syndrome (Batla et al. 2015).
p.R232H/p.H277L + p.T251I/p.P587L	26–40	Dysphagia, Proximal muscle weakness, Migraine, SANDO syndrome (Batla et al. 2015)
p.Y986D + p.M919T	34	Migraine, Depression, SANDO syndrome (Batla et al. 2015)
p.A467T + p.R1138C	35‐46F	Dysphagia, Proximal muscle weakness, Exercise intolerance, Chorea, Depression, SANDO syndrome (Batla et al. 2015; Miguel et al. 2014).
p.A467T + p.L559P	50	Dysphagia, Proximal muscle weakness, SANDO syndrome (Miguel et al. 2014).
p.A467T + p.G848S	NA	SANDO syndrome (Batla et al. 2015).
p.P587L + p.T251I/p.R869Q	29F	Proximal muscle weakness, SANDO syndrome (Weiss and Saneto 2010).
p.L304R + p.L304R	15M	SANDO syndrome (Lovan et al. 2013).
p.P648R + p.R807C	39M	Dysphagia, SANDO syndrome (Bandettini di Poggio et al. 2013).
p.T251I + p.P587L/p.A467T	55F	CD, Resting tremor, Depression, Diabetes, Rectal incontinence, Proximal muscle weakness, SANDO syndrome (Miguel et al. 2014).
p.A899T + p.A899T	26F	Parkinsonism, CD, Anxiety and obsessive disorder, Proximal muscle weakness, SANDO syndrome (Stumpf and Copeland 2011).
p.L592F + p.R1096C	38F	Dysphagia, RLS, SANDO syndrome (Hanisch et al. 2015).
p.P765T + p.P765T	42F	Gastroparesis, Optic discs atrophy, Anorexia, SANDO syndrome (Weiss and Saneto 2010).
p.T251I + p.P587L/p.G848S	73M	Proximal muscle weakness, SANDO syndrome (Richter et al. 2018).
p.A467T/p.W748S + p.E1143G	37M	SANDO syndrome (Milone et al. 2008).
p.T251I + p.G848S	50M	Dysphagia, SANDO syndrome (Milone et al. 2008).
p.W748S/p.E1143G + p.W748S/p.E1143G	27F‐32M	Proximal muscle weakness, Hypogonadotropic hypogonadism, Dysphagia, Tremor, SANDO syndrome (Horvath 2006).
p.A467T + p.G737R	38M	Dysphagia, Constipation, CD, Hearing loss, Muscle cramps, SANDO syndrome (Horvath 2006).
p.A467T + p.E1143G	15M	Proximal muscle weakness, Dysphagia, CD, SANDO syndrome (Rajakulendran et al. 2016).
p.A467T + p.A467T	5M‐16F‐17F‐20M‐31F‐41M‐42F	Epilepsy, CD, Visual symptom, Tremor, Dysphagia, Proximal muscle weakness, Impotence, Socially Withdrawn, Dysphagia, 2 Diminished visual acuity, SANDO syndrome (Richter et al. 2018; Milone et al. 2008; Horvath 2006) (Mancuso et al. 2004; Van Goethem et al. 2003).
p.W748S + p.Q497H	23M	Myoclonus, Depression, SANDO syndrome (Van Goethem et al. 2003).
p.A467T + p.W748S/p.E1143G	12M	Tremor, SANDO syndrome (Van Goethem et al. 2003).
p.H932T + p.G1051R	18M‐20F	Hearing loss, CD, Depression, Right bundle branch block, SANDO syndrome (Tanaka et al. 2013).
p.A467T + p.L304R	25F	Depression, SANDO syndrome.
p.A467T + p.R3P	20M‐30F	Proximal muscle weakness, Dysphagia, SANDO syndrome (Tanaka et al. 2013).
p.A467T + p.R627W	19M‐32M	Proximal muscle weakness, Dysphagia, Cardiomyopathy, Hearing loss, SANDO syndrome (Van Goethem et al. 2003).
p.L316L + p.A1217V	54M	Difficulty hearing, SANDO syndrome (Tanaka et al. 2013).
p.S998L + p.I898T	16F	Tremor, cognitive deficits, hypomimia, hypopsia, SANDO syndrome (Ma et al. 2020).

Abbreviations: AAO, age‐at‐onset; CD, cognitive defect; F, female; M, male; NA, not available; RLS, restless leg syndrome; SANDO syndrome, Sensory ataxic neuropathy, dysarthria, and ophthalmoparesis.

The mean age of onset for POLG‐related SANDO was 31.6 years (range: 5–73 years), with 95.1% of patients presenting symptoms prior to the age of 50. In our retrospective analysis, most patients exhibited the classic SANDO triad, while approximately 6.5% presented with sensory ataxia and ophthalmoparesis without dysarthria. Apart from the triad of symptoms of SANDO syndrome, other clinical manifestations include: proximal muscle weakness (28.5%), dysphagia (23.7%), cognitive defect (24.0%), depression (14.3%), tremor (11.3%), parkinsonism (9.5%), migraine (6.4%), and so on.

## 4 Discussion

The POLG gene, located on chromosome 15q25, encodes the catalytic subunit of DNA Pol γ, the enzyme responsible for mtDNA replication and repair (Rahman and Copeland 2019). Mutations in POLG are associated with a spectrum of disorders, including Alpers syndrome, Alpers‐like encephalopathy, childhood myocerebrohepatopathy spectrum disorders, ataxia neuropathy spectrum, and SANDO syndrome (Cohen et al. 1993).

In this case, SANDO syndrome was caused by two variants in the POLG gene: c.3297G>C (p.W1099C) and c.1774C>T (p.L592F). The clinical manifestations included dysarthria, external ophthalmoplegia (bilateral ptosis and restricted eye movements in all directions), peripheral neuropathy (reduced distal tendon reflexes in the lower limbs and diminished deep and superficial sensation in both lower extremities), and ataxia (positive Romberg sign and abnormal gait).

Both variants are located in critical functional regions of the POLG protein. Mutations in POLG lead to mitochondrial dysfunction, which underlies the corresponding clinical features. The p.L592F variant has been previously reported in patients with SANDO syndrome, who also exhibited sensory ataxic neuropathy, dysarthria, ophthalmoplegia, and dysphagia (Kurt et al. 2012).

It remains unclear whether the clinical manifestations in this patient primarily result from reduced polymerase activity caused by one of the variants, from the combined effects of both variants, or which of the two mutations has a greater impact on polymerase activity.

In our report, only mutations in the POLG gene were found; no single or multiple mtDNA deletions or other mutations were detected. According to previous case reports, SANDO syndrome can be associated with large‐scale mtDNA deletions. It is considered a subtype of CPEO with multiple mtDNA deletions. In a retrospective analysis of 107 mitochondrial myopathy cases featuring single or multiple mtDNA deletions, none of the patients with single, large‐scale deletions met the clinical criteria for SANDO syndrome. Among the 41 cases with multiple mtDNA deletions, approximately 22% were diagnosed with SANDO, and 67% of this subgroup carried nuclear POLG mutations. This suggests that SANDO syndrome represents the most frequent phenotype linked to multiple mtDNA deletions and often co‐occurs with POLG mutations, whereas it is rarely associated with single, large‐scale mtDNA deletions (Hanisch et al. 2015). Whether a correlation exists between mtDNA deletions and POLG mutations remains to be established through large‐scale, systematic studies.

Our analysis revealed that the most frequent POLG variants were p.A467T and p.W748S, which is consistent with previous reports indicating that these are the most common POLG mutations in European populations (Anagnostou et al. 2016; Han et al. 2017). However, the variant identified in our patient is not a common variant in European populations, large‐scale epidemiological data about POLG are still lacking in China.

A growing body of evidence indicates that molecules such as PNPT1, TRMU, LRPPRC, and TFAM, though not directly involved in mtDNA replication, play critical roles in mitochondrial RNA processing, modification, translation, and stability. Defects in these processes can impair mitochondrial protein synthesis, leading to clinical manifestations that closely overlap with those caused by POLG mutations.

For instance, LRPPRC encodes an approximately 158 kDa RNA‐binding protein localized predominantly in mitochondria. It forms a heterodimer with SLIRP to protect mitochondrial mRNAs from degradation, extend their half‐life, and promote polyadenylation, thereby supporting efficient translation. Mutations in LRPPRC are associated with Leigh syndrome, which presents with infantile‐onset progressive neurodegeneration, lactic acidosis, and brainstem lesions—features that mirror phenotypes observed in POLG deficiency (Parada‐Garza et al. 2020).

In summary, while POLG dysfunction impairs mtDNA replication and reduces transcription templates, mutations in mtRNA stability factors—such as LRPPRC, PNPT1, and SLIRP—shorten mRNA half‐life or disrupt translation initiation, diminishing transcript utilization. Both mechanisms converge to cause OXPHOS failure, lactic acidosis, reduced ATP production, and elevated ROS levels. This shared pathophysiology underlies the frequently indistinguishable clinical and pathological phenotypes seen in POLG‐related and mtRNA stability‐related disorders.

Current treatment options for mitochondrial myopathy remain limited. The patient was managed with a mitochondrial cocktail (idebenone, coenzyme Q10, vitamins B and E) and exercise therapy. Follow‐up assessments indicated mild symptomatic improvement, though she remained unable to resume normal occupational activities.

Furthermore, we comprehensively reviewed all reported POLG mutations associated with SANDO syndrome and their clinical characteristics. These results provide valuable insights for early diagnosis and recognition of SANDO syndrome in clinical practice.

## Author Contributions

**Fanjing Zhou**: conceptualization, data curation, investigation, validation, writing – review and editing, writing – original draft. **Jiang Chen**: conceptualization, data curation, formal analysis, investigation, methodology, writing – original draft. **Tingzheng Zhang**: conceptualization, data curation, investigation, methodology, writing – review and editing. **Fengnan Niu**: data curation, validation, writing – review and editing. **Jinglong Hu**: data curation, investigation, writing – review and editing. **Yun Xu**: data curation, methodology, writing – review and editing. **Meijuan Zhang**: conceptualization, data curation, formal analysis, investigation, methodology, project administration, supervision, writing – original draft.

## Funding

This study was supported by the National Natural Science Foundation of China (Grant Numbers 82471352, 82071408, and 81920108017), the National Key Research and Development Program of China (Grant Number 2023ZD0505200), the Natural Science Foundation of Jiangsu Province (Grant Number BK20191116).

## Ethics Statement

We confirm that we have read the Journal's position on issues involved in ethical publication and affirm that this report is consistent with those guidelines.

## Consent

Consent for publication was obtained from the patient.

## Conflicts of Interest

The authors declare no conflicts of interest.

## Supporting information




**Supporting Fig.1: Immunohistochemical staining of the patient's nerve biopsy**. The figure showed that there was no obvious infiltration of CD4, CD8 and CD20 positive cells in the neural tissue (first row). Positive cell control diagram (the second row).

**Supporting Fig.2: The efficacy of virus transfection**. qRT‐PCR was used to detect the expression of wild‐type POLG. Compared with the empty vector group, the expression in the mPOLG knockout group significantly decreased, proving the effectiveness of the knockout system. *p<0.05.

## Data Availability

All relevant data are contained within the manuscript.
