# Supplementary figures and images for: Characterization of Novel POLG Mutations in Mitochondrial Encephalomyopathy: Pathogenic Validation and Comprehensive Genetic Profiling
Source: Brain Behav. 2025 Nov 11;15(11):e71045. doi: 10.1002/brb3.71045 (PMC12605960; doi:10.1002/brb3.71045)

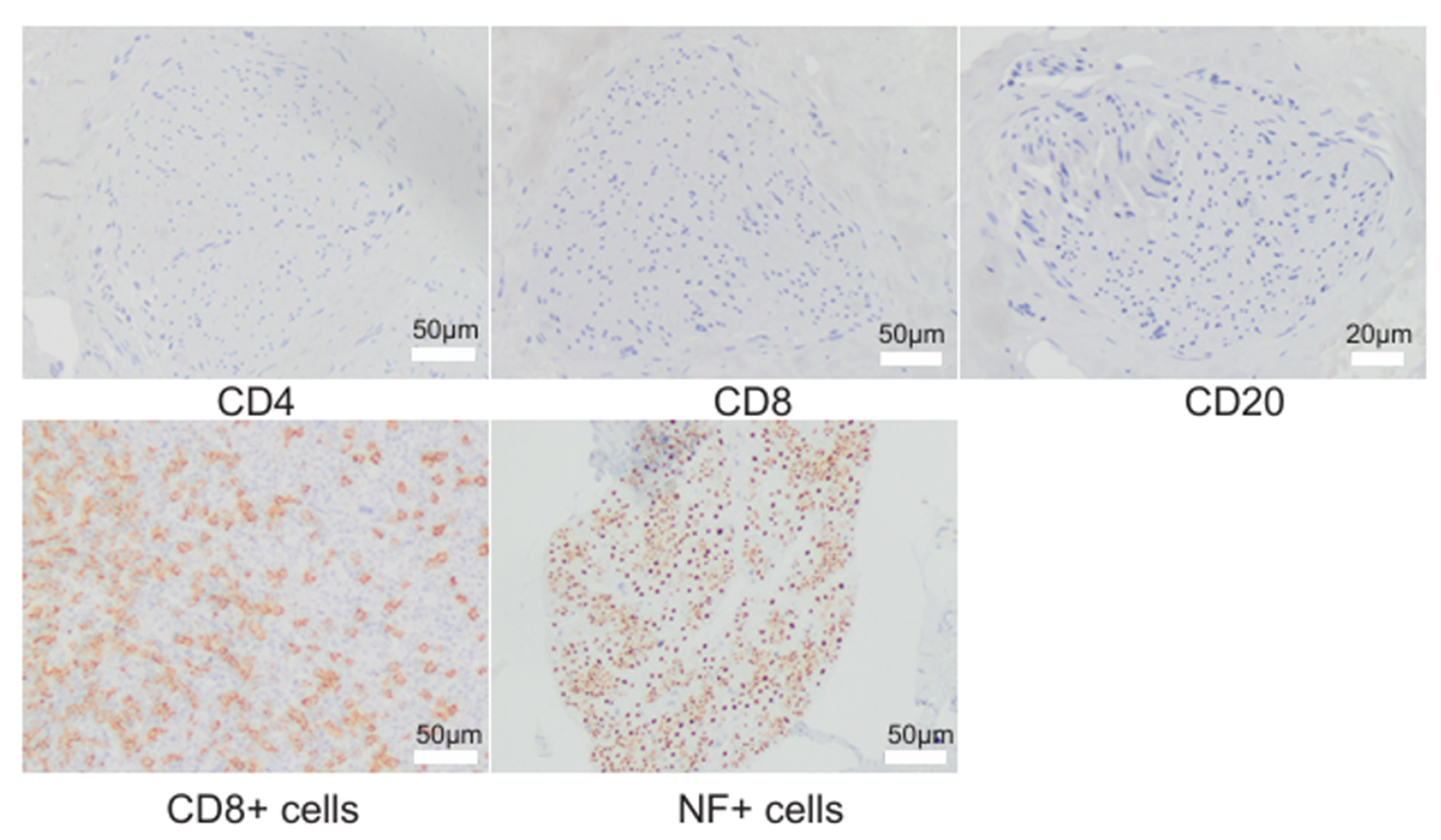

Supplement: Supplementary file 1 — Supporting Fig.1: Immunohistochemical staining of the patient's nerve biopsy. The figure showed that there was no obvious infiltration of CD4, CD8 and CD20 positive cells in the neural tissue (first row). Positive cell control diagram (the second row). [file BRB3-15-e71045-s001.tif]

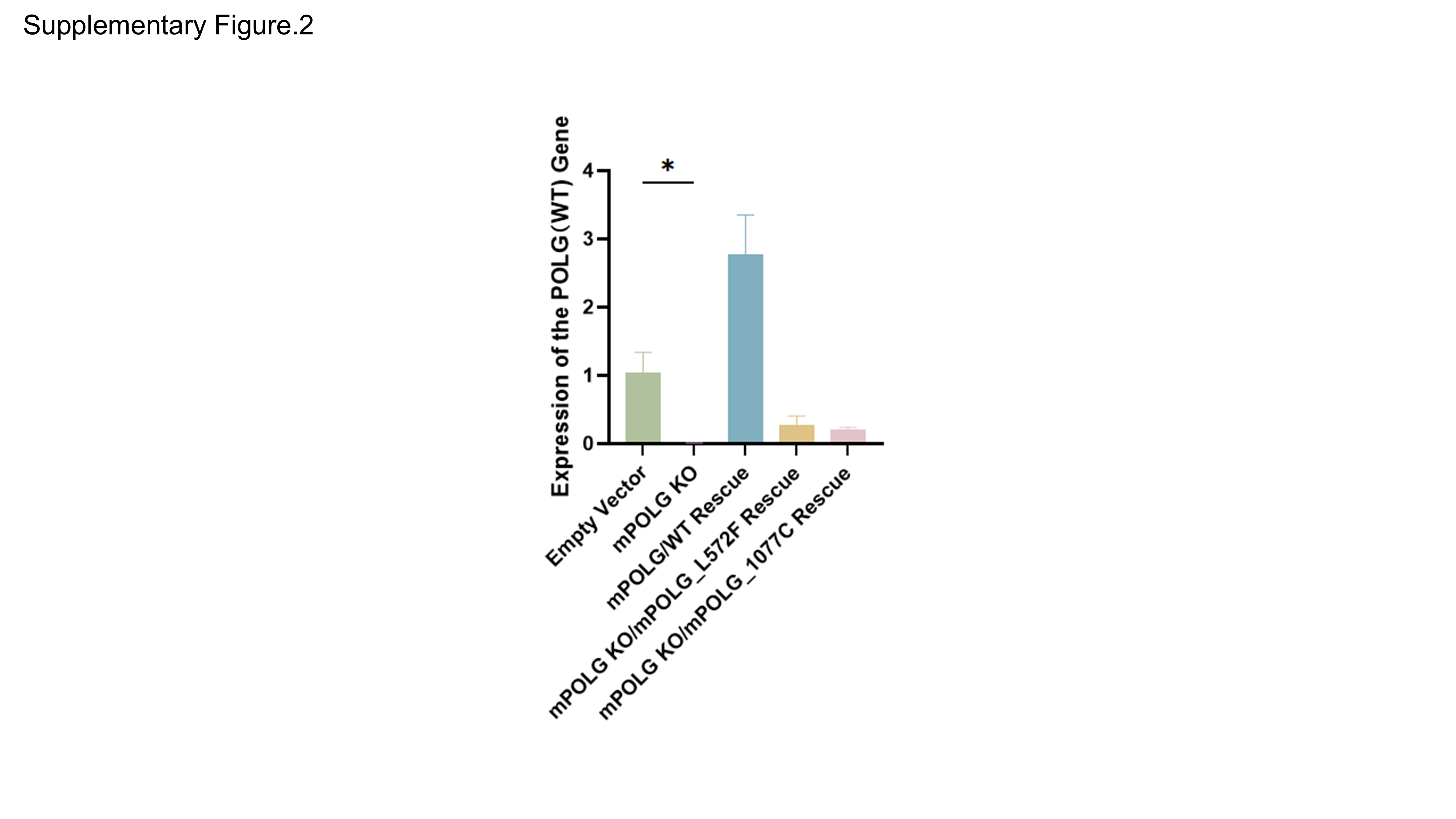

Supplement: Supplementary file 2 — Supporting Fig.2: The efficacy of virus transfection. qRT‐PCR was used to detect the expression of wild‐type POLG. Compared with the empty vector group, the expression in the mPOLG knockout group significantly decreased, proving the effectiveness of the knockout system. *p<0.05. [file BRB3-15-e71045-s002.tif]
